# Testing the Efficacy of Training Basic Numerical Cognition and Transfer Effects to Improvement in Children’s Math Ability

**DOI:** 10.3389/fpsyg.2018.01775

**Published:** 2018-10-02

**Authors:** Narae Kim, Selim Jang, Soohyun Cho

**Affiliations:** ^1^Department of Psychology, Chung-Ang University, Seoul, South Korea; ^2^Department of Psychology, University of Illinois at Urbana-Champaign, Champaign, IL, United States

**Keywords:** approximate number sense, training, numerosity comparison, numberline estimation, approximate arithmetic, symbol-to-numerosity mapping

## Abstract

The goals of the present study were to test whether (and which) basic numerical abilities can be improved with training and whether training effects transfer to improvement in children’s math achievement. The literature is mixed with evidence that does or does not substantiate the efficacy of training basic numerical ability. In the present study, we developed a child-friendly software named “123 Bakery” which includes four training modules; non-symbolic numerosity comparison, non-symbolic numerosity estimation, approximate arithmetic, and symbol-to-numerosity mapping. Fifty-six first graders were randomly assigned to either the training or control group. The training group participated in 6 weeks of training (5 times a week, 30 minutes per day). All participants underwent pre- and post-training assessment of their basic numerical processing ability (including numerosity discrimination acuity, symbolic/non-symbolic magnitude estimation, approximate arithmetic, and symbol-to-numerosity mapping), overall math achievement and intelligence, 6 weeks apart. The acuity for numerosity discrimination (approximate number sense acuity; hereafter ANS acuity) significantly improved after training, but this training effect did not transfer to improvement in symbolic, exact calculation, or any other math ability. We conclude that basic numerical cognition training leads to improvement in ANS acuity, but whether this effect transfers to symbolic math ability remains to be further tested.

## Introduction

The ability to process numerosity information is essential for everyday life in both humans and animals (Agrillo et al., 2008; Libertus et al., 2011; Leibovich et al., 2017). Approximate number sense (ANS) enables the ability to grasp approximately how many items there are and to roughly add or subtract sets of items. Some researchers believe that basic numerical processing ability and higher level mathematical achievement builds on the ANS (Rousselle and Noël, 2007; De Smedt and Gilmore, 2011; Sasanguie et al., 2012, 2013; Jang and Cho, 2018). Basic numerical processing includes numerosity comparison, symbolic number comparison, numberline estimation, and understanding the mapping between symbolic numbers and their corresponding numerosity (or non-symbolic magnitude), etc. Basic numerical processing abilities are reported to predict future math achievement (Jordan et al., 2007; Locuniak and Jordan, 2008; Lyons and Beilock, 2011; Mazzocco et al., 2011; Sasanguie et al., 2013; Starr et al., 2013; Martin et al., 2014). Furthermore, children with mathematical learning disabilities or developmental dyscalculia have been found to show low performance on basic numerical processing (Rousselle and Noël, 2007; Geary et al., 2008; De Smedt et al., 2009; Piazza et al., 2010; De Smedt and Gilmore, 2011). Some studies reported that training on basic numerical abilities led to improvement in math achievement (Park and Brannon, 2013, 2014; Park et al., 2016; Sella et al., 2016). However, different types of training were used across studies and the reports of the efficacy of training were mixed. Thus, at present it is not easy to draw a conclusion on whether or not intervention on basic numerical abilities can improve one’s math performance (Schneider et al., 2016; Szűcs and Myers, 2017).

In some studies, training on approximate arithmetic (approximate addition and subtraction) using dot arrays improved the training groups’ symbolic addition/subtraction abilities compared to the control group (Park and Brannon, 2013, 2014; Hyde et al., 2014; Khanum et al., 2016; Park et al., 2016; Au et al., 2018; Szkudlarek and Brannon, 2018). In contrast, Räsänen et al. (2009) did not find any improvement on arithmetic (addition and subtraction) and counting abilities after training with the Number Race program^1^ (Wilson et al., 2006) although children’s ANS acuity was improved (Räsänen et al., 2009). Szkudlarek and Brannon (2018) reported that approximate arithmetic vs. numeral identification training was effective for preschoolers with low vs. high math skills, respectively. But the training improved only early informal, but not formal, math skills. Based on a meta analysis, Szűcs and Myers (2017) concluded that, presently, there is no evidence that ANS training improves symbolic arithmetic given methodological issues and heterogeneity across studies. One crucial issue relates to the inclusion of symbolic arithmetic practice within the training program itself (Wilson et al., 2006, 2009; Räsänen et al., 2009; Vilette et al., 2010; Kucian et al., 2011; Obersteiner et al., 2013; Sella et al., 2016). In these cases, improvement in symbolic math ability after repeated practice of symbolic arithmetic may simply reflect practice (or test–retest) effect rather than a true transfer effect of training. Furthermore, many studies reporting a significant training effect tended to have small effect sizes or unstable results (e.g., being influenced by outliers) (Szűcs and Myers, 2017).

The goal of the present study was to investigate whether (and which) basic numerical abilities can be improved with training and to test whether the training effect transfers to improvement in overall math achievement. We developed a child-friendly computer based software named “123 Bakery” which included four modules for training basic numerical abilities (numerosity comparison, numberline estimation, approximate, non-symbolic addition/subtraction, and symbol-to-numerosity mapping). Exact, symbolic arithmetic practice during training was purposefully excluded in order to thoroughly test whether the effect of training on basic numerical cognition truly transfers to exact, symbolic math ability without explicit practice in this domain. Our software was designed to include several training modules within each session, as in typical educational interventions (Kroesbergen and Van Luit, 2003; Gersten et al., 2009; Codding et al., 2011). Training sessions were administered at the child’s home which increased ecological validity of our training to real-world educational applications. All assessments were administered at the child’s home as well. The difficulty level of training was tailored to each participant to help participants learn in their own zone of proximal development (i.e., adaptive training). Training effects were tested by comparing assessment scores acquired immediately before and after training. In order to measure improvement of trained abilities while minimizing test–retest effects, we designed tasks with alternative visual interfaces (see Materials and Methods for details). Mathematical achievement was assessed with a comprehensive standardized math test battery (which included number concept, arithmetic, geometry, and problem solving) and a computerized arithmetic test. We induced intensive home training over 6weeks (5 days/week, 35 min/day) which is by far the longest in the total duration of training compared to previous studies. The range of numerosity was also much extended (up to 300 depending on performance) which was much larger than most previous studies (which included numerosities up to 80) (Wilson et al., 2006, 2009; Räsänen et al., 2009; Kucian et al., 2011; Park and Brannon, 2013, 2014; Park and Brannon, 2014; Hyde et al., 2014; Park et al., 2016; Sella et al., 2016; Au et al., 2018). In other words, the present study aimed to rigorously test the efficacy of training basic numerical abilities based on sufficiently long durations across a large range of magnitudes while minimizing the influence of test–retest effects. The duration of training in the present study was longer than that of other lab-based training studies (but it was similar to the average duration of intervention/training programs commonly used in real-world educational settings (Cohen et al., 1982; Kroesbergen and Van Luit, 2003). Furthermore, we carefully controlled for non-numerical visual properties of the non-symbolic stimuli (dot arrays) during training and assessment, so that the influence of non-numerical visual magnitudes can be minimized. Finally, our home-based training procedure improved the ecological validity of our training program enabling more confident generalization to real-world, educational applications compared to studies which conducted training in lab settings (Hyde et al., 2014).

Given inconsistencies in the literature, we did not have an *a priori* hypothesis in favor of the idea that basic math abilities can be improved with training and that such training effects will be transferred to improvement in math achievement (especially when exact calculation is not included in the training). By using sufficiently long duration of training and wide range of magnitudes (while controlling for the influence of non-numerical visual magnitudes), we did not make type II error due to insufficient duration/range of training or contamination by extraneous variables.

## Materials and Methods

### Participants

Fifty-six 1st graders participated in the study. Data from 10 children who did not complete the experiment or whose performance (on pre-training numerosity comparison or the final level reached on the training modules) was lower than 2 SDs below the mean were excluded. (See **Supplementary Materials** for further details.) Thus, data from forty-six children were included in the analysis (24 females; mean age = 7.70 years; and *SD* = 0.30). Participants were recruited by advertisement. All participants and their parents provided written informed consent before participation. The IRB committee of Chung-Ang University approved all protocols of the study (IRB-2013-55). Participants were randomly assigned to either the training or control group. Participants received monetary compensation after completion of the experiment.

### Procedure

**FIGURE 1:**
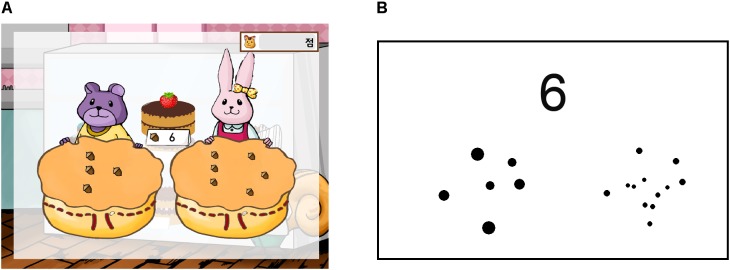
Screenshots of an example trial of **(A)** the “Selling Cakes” module of “123 Bakery” and **(B)** “Symbol-to-Numerosity Mapping” task used for pre- and post-training assessment.

Participants were randomly assigned to either the training (*n* = 22) or control (*n* = 24) group. Pre-training assessments [including basic numerical processing tasks, two math achievement tests, and the Raven’s Advanced Progressive Matrices (APMs) test] were administered to all participants. Only the training group participated in 30 training sessions over 6 weeks using a computerized software (“123 Bakery”). After 6 weeks had passed since the administration of the pre-training assessment, all participants were administered post-training assessment. The pre- and post-training assessments of basic numerical abilities were conducted with four tasks corresponding to each training module using alternate visual formats in order to minimize practice or test–retest effects at the visuomotor level (see **Figure 1**).

### Materials

#### Basic Numerical Cognition Training Program “123 Bakery”

**FIGURE 2:**
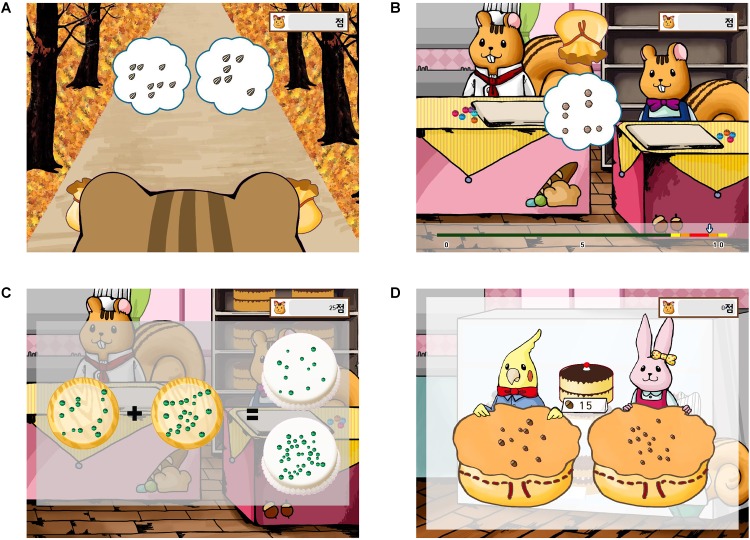
Screenshots of example trials from **(A)** “Gathering Ingredients,” **(B)** “Guess How Many?”, **(C)** “Cake Decoration,” and **(D)** “Selling Cakes” modules of “123 Bakery.”

We developed a computerized program named “123 Bakery” which composed of four training modules. The four training modules included (1) numerosity comparison (“Gathering Ingredients”), (2) non-symbolic numberline estimation (“Guess How Many?”), (3) Approximate Addition & Subtraction (“Cake Decoration”), and (4) Symbol-to-Numerosity Mapping (“Selling Cakes”). (Each training module is explained in the next section.) Each module was 6 minutes long. Feedback on the correctness of the response was provided after each trial. The cumulative total score (within each session) was updated real-time and was always shown on the top right-hand side of the screen (**Figure 2**). Task difficulty increased as subjects mastered each Level by accomplishing a certain degree of performance accuracy (0.7–0.9 accuracy among the last 10–20 trials depending on the Level; see **Supplementary Tables S1**–**S4** for details).

In order to control for the influence of non-numerical visual properties of dot arrays (e.g., individual dot size, cumulative surface area, and convex hull) during numerosity processing, we made convex hull equivalent for all dot arrays and divided trials into two control conditions (area vs. size controlled conditions) (Pica et al., 2004; Halberda and Feigenson, 2008; Jang and Cho, 2016; Park and Cho, 2016; Lee and Cho, 2017). First, on half of the trials, dot arrays were matched on cumulative surface area (area controlled condition) and on the other half of the trials, dot arrays were matched on individual dot size (size controlled condition). The order of trial presentation was randomly intermixed. Although it is not possible to perfectly control for the influence of non-numerical visual properties of dot arrays during numerosity processing, the use of randomly intermixed control conditions and making convex hull equivalent across all dot arrays ensured that non-numerical visual magnitude could not be reliably used as an alternative cue to guess numerosity (Maloney et al., 2010; Gebuis and Reynvoet, 2012a,b; Leibovich and Henik, 2013; Dietrich et al., 2015).

##### Training module 1: numerosity comparison (“gathering ingredients”)

Two arrays (of berries or nuts) appeared side by side for 1,000 ms. Subjects were instructed to choose the more numerous array (**Figure 2A**). Task difficulty increased as the set size became larger (range = 6–200) and as the ratio of magnitudes approached 1 (range = 2:3–9:10). Audiovisual feedback on the correctness of the response was provided after each trial.

##### Training module 2: non-symbolic Numberline Estimation (“Guess How Many?”)

Subjects were presented with an array (of berries or nuts, etc.) for 1,000 ms at the center of the screen. Subjects were asked to click on a location on the numberline which corresponds to the estimated numerosity of the elements of the array (**Figure 2B**). If the estimate was within the “accurate zone” (see **Supplementary Table S2** for details), positive feedback was given. Task difficulty varied by the numerosity of the stimulus, the maximum value (end point) of the numberline, and the relative width (i.e., proportion) of the accurate zone.

##### Training module 3: non-symbolic addition/subtraction (“cake decoration”)

Subjects were presented with two arrays (of berries or nuts, etc.) for 1,000 ms and were asked to perform approximate addition or subtraction. Then, two arrays were additionally shown as options to choose from. Subjects were asked to respond by choosing one of the two options which seemed closer to their approximate answer within 6 s (**Figure 2C**). Audiovisual feedback on the correctness of the response was provided after each trial.

##### Training module 4: symbol-to-numerosity mapping (“selling cakes”)

Subjects were asked to choose which animal character (the customer) possessed the correct number of nuts which corresponded to the price of the cake (shown as a numeral at the center of the screen) (**Figure 2D**). Task difficulty increased as the ratio of numerosities approached 1 and as the price of the cake increased. Audiovisual feedback on the correctness of the response was provided after each trial.

#### Pre- and Post-training Assessments

##### Four basic numerical processing tasks

The four basic numerical processing tasks (numerosity comparison, symbolic and non-symbolic numberline estimation, approximate arithmetic, and symbol-to-numerosity mapping) had the same structure as the four training modules except that the array consisted of black dots on a white background. Each task is explained in the following sections.

###### Numerosity comparison

**FIGURE 3:**
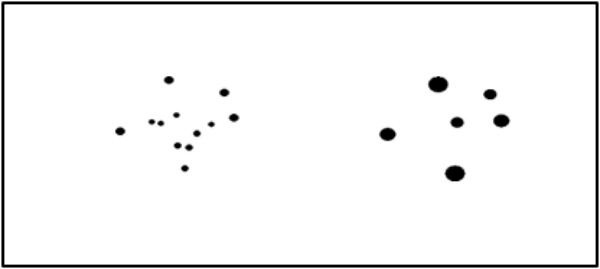
Example trial of the numerosity comparison task.

Subjects were presented with a pair of dot arrays (1,000 ms) and were asked to choose the array with the greater number of dots (**Figure 3**). Subjects pressed the #3 key for the array on the left and #8 key for the array on the right. The left–right location of the correct answer was counterbalanced. The ratio of numerosities included 1:2, 3:4, 5:6, 6:7, 7:, and 8:9. The entire stimulus list is shown in **Supplementary Table S5**. There was a total of 120 trials.

###### Symbolic and non-symbolic numberline estimation

**FIGURE 4:**
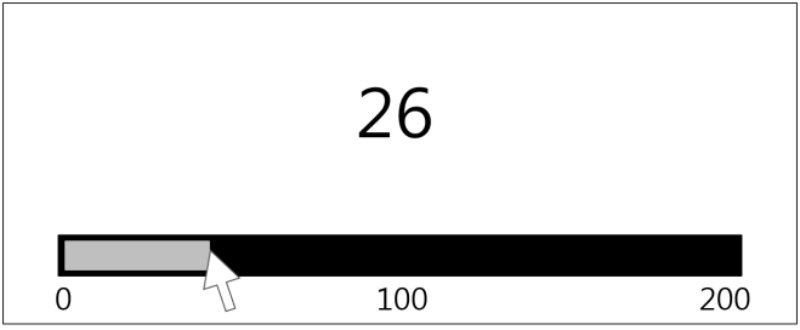
Example trial of the symbolic numberline estimation task with an endpoint of 200.

The numberline estimation task was conducted using both symbolic (Arabic numerals) and non-symbolic magnitudes (**Figure 4**). The trials were divided into two blocks based on the value of the end point of the numberline (100 or 200). The stimuli included 5, 18, 32, 55, 73, and 98 for block 1 and 5, 18, 42, 78, 111, 133, 147, 172, and 187 for block 2. The target stimulus appeared for 1,000 ms.

The accuracy of performance was calculated with Percent Absolute Error (PAE; Eq. 1) (Siegler and Booth, 2004). Smaller PAE represents smaller error in estimation and greater linearity in mental magnitude representations (Siegler and Booth, 2004; Booth and Siegler, 2006). For each target stimulus, three trials were repeated. The mean PAE for each target stimulus was used as the dependent variable (Siegler and Ramani, 2009).

$$\mathrm{PAE}=\frac{\mathrm{Estimate}-\mathrm{Estimated Magnitude}}{\mathrm{The scale of the Numberline}}$$

Eq. 1. Calculation of PAE

###### Approximate arithmetic

**FIGURE 5:**
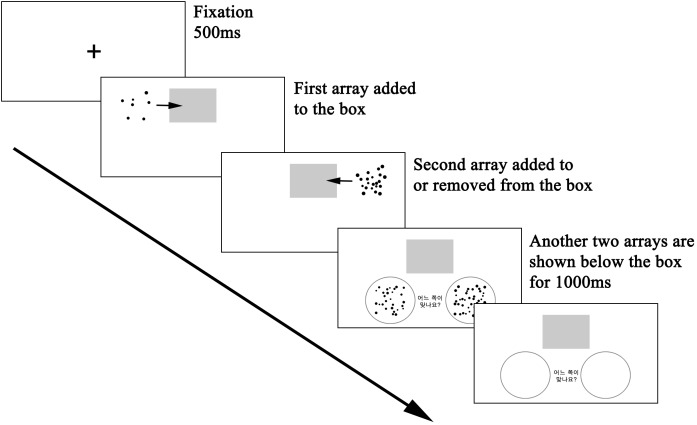
Example trial procedure of the approximate arithmetic task.

The procedure of the task was similar to the Approximate Arithmetic condition used in Park and Brannon (2013, 2014). This task was administered in two separate blocks for addition and subtraction. Subjects were first shown a dot array which was added to a gray box (**Figure 5**). Next, another dot array was either added to or removed from the gray box. Finally, the subjects chose one of two new dot arrays whose numerosity seemed closer to the perceived total number of dots in the gray box. They responded by pressing the #3 key to choose the array on the left and #8 key for the array on the right. Task difficulty was manipulated by the ratio of the set sizes of the two arrays (4:5, 4:6, and 4:7) presented as a pair on each trial. Addition and subtraction were performed on arrays with numerosities ranging from 6–51. The numerosity of arrays presented as options ranged from 16–91. Including 5 practice trials, a total of 35 trials were administered.

###### Symbol-to-numerosity mapping

Subjects were asked to choose one of two arrays presented for 1000 ms whose numerosity matched the Arabic number presented at the center of the screen (**Figure 1B**). The ratio between the magnitude of the stimuli varied from 1:1.75 to 4:5 (1:1.75, 1:2, 2:3, 3.5:5, 3:4, and 4:5). The set size of the stimuli varied from 6 to 100 (6–30, 30–50, and 50–100). The left–right position of the correct answer was counterbalanced. A total of 144 trials were administered. The order of trials from each ratio/condition was randomly intermixed.

#### Mathematical Achievement Tests

##### Comprehensive math achievement test (KNISE-BAAT)

The Korean National Intelligence for Special Education–Basic Academic Achievement Test (KNISE-BAAT for math) (Park et al., 2008) was used to measure mathematical performance. KNISE-BAAT consists of four subdomains (number concept, arithmetic, geometry, and problem solving).

##### Computerized arithmetic task

Subjects solved 64 problems of addition and subtraction without paper and pencil on a computer. Three ranges of numbers were used (6–30, 30–50, and 50–99). Participants were instructed to type the answer using the number keys on the keyboard. There was no time limit. (Accuracy rather than RT of problem solving was emphasized). Thus, accuracy rather than RT was the main dependent variable of interest.

##### Raven’s APM test

Children’s fluid intelligence was measured with an abbreviated version of the Raven’s APM test (Arthur et al., 1999). This score was used as a covariate in order to control individual differences in fluid intelligence.

## Results

### Test of Between-Group Differences in Pre/Post-training Assessments

The training and control group were matched on age and gender. Independent samples *t*-tests revealed no difference in age [*t*(44) = 1.99, *p* = 0.05] and gender [*t*(44) = 0.86, *p* = 0.39] between groups. In addition, our groups did not differ on pre- and post-training assessments of math achievement or fluid intelligence (*p*s > 0.05; **Table 1**).

**Table 1:** Descriptive statistics of performance from the pre- and post-training assessments of basic numerical processing abilities.

		Pre-training			Post-training		
		Training	Control	*t*-test	Training	Control	*t*-test
Numerosity comparison	ACC	0.75 (0.07)	0.72 (0.09)	1.30 (*p* = 0.20)	0.81 (0.07)	0.71 (0.08)	4.37 (*p* < 0.001)
	RT	1,532.02 (315.04)	1,569.41 (286.59)	−42 (*p* = 0.68)	1,460.85 (345.24)	1,428.15 (304.51)	0.34 (*p* = 0.73)
Symbolic numberline estimation	PAE	0.05 (0.02)	0.05 (0.02)	−0.22 (*p* = 0.82)	0.05 (0.01)	0.05 (0.02)	−1.51 (*p* = 0.14)
	RT	3,706.75 (1,316.72)	3,818.01 (1,221.40)	−0.30 (*p* = 0.77)	2,710.71 (623.46)	3,576.84 (993.24)	−3.50 (*p* < 0.001)
Non-symbolic numberline estimation	PAE	0.14 (0.03)	0.14 (0.04)	−0.22 (*p* = 0.83)	0.16 (0.05)	0.14 (0.03)	1.78 (*p* = 0.08)
	RT	2,549.57 (881.61)	2,650.57 (1,085.01)	−0.35 (*p* = 0.73)	2,664.78 (814.05)	2,772.19 (1,023.36)	−0.39 (*p* = 0.70)
Non-symbolic addition	ACC	0.60 (0.10)	0.56 (0.08)	1.38 (*p* = 0.18)	0.59 (0.08)	0.59 (0.08)	−0.10 (*p* = 0.93)
	RT	1,959.34 (763.18)	2,150.39 (920.51)	−0.76 (*p* = 0.45)	2,053.98 (938.94)	2,028.04 (613.66)	0.11 (*p* = 0.91)
Non-symbolic subtraction	ACC	0.53 (0.08)	0.50 (0.07)	1.11 (*p* = 0.28)	0.55 (0.06)	0.54 (0.09)	0.47 (*p* = 0.64)
	RT	1,933.03 (619.19)	2,221.99 (836.18)	−1.32 (*p* = 0.19)	2,305.66 (949.68)	2,476.66 (2,220.41)	−0.33 (*p* = 0.74)
Symbol-to-numerosity mapping	ACC	0.60 (0.06)	0.60 (0.06)	0.23 (*p* = 0.82)	0.61 (0.06)	0.58 (0.07)	1.33 (*p* = 0.19)
	RT	1,120.88 (290.35)	1,477.01 (906.20)	−1.76 (*p* = 0.09)	1,165.01 (304.71)	1,339.36 (0.377.88)	−1.71 (*p* = 0.09)
Raven’s APM		0.43 (0.19)	0.48 (0.18)	0.81 (*p* = 0.43)	0.55 (0.19)	0.48 (0.19)	1.227 (*p* = 0.23)

*Numbers in parentheses denote SDs. The results of between-group t-tests at each time (pre-training and post-training) for each assessment scores are also shown.*

### Training Effects on Basic Number Processing Abilities

**FIGURE 6:**
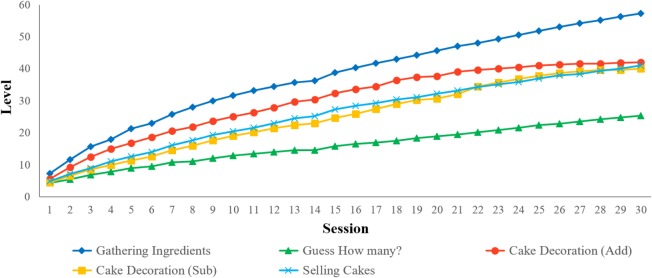
Participants’ average of the level reached at the end of each session for each module of “123 Bakery.”

**FIGURE 7:**
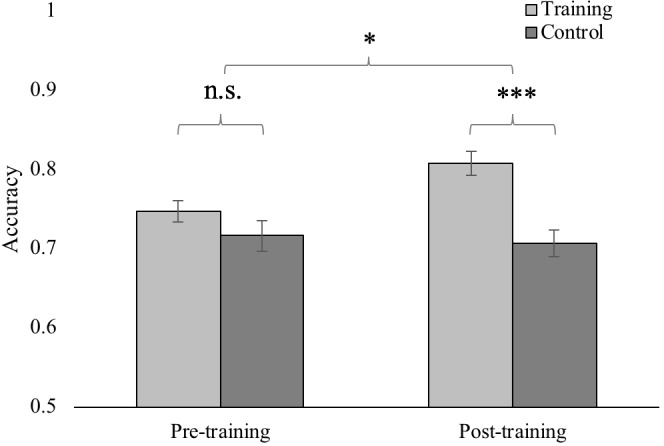
Pre- and post-training assessment of numerosity comparison accuracy of the training and control groups. Bars represent SEM. A 2×2 mixed repeated measures ANOVA manifested a significant two way interaction between time and group. *Post hoc* *t*-tests revealed that the group difference in numerosity comparison accuracy was significant only at post-training. (^∗^*p* < 0.05, ^∗∗∗^*p* < 0.001, n.s. = not significant)

Descriptive statistics of performance from pre- and post- training assessments of basic number processing abilities are provided in **Table 1**. The training group’s performance at the end of each session for each module of “123 Bakery” are shown in **Figure 6**. The final Level reached at the end of training for each module of “123 Bakery” and the overall average of the 30 mean performance scores (accuracy and RT) from each session are provided in **Supplementary Table S6**. In order to test for training effects, a 2×2 mixed repeated measures ANOVA was conducted on basic number processing performance (numerosity comparison, symbolic and non-symbolic numberline estimation, symbol-to-numerosity mapping, and approximate arithmetic) with time (pre-, post-training) as the within-subject factor and group (training, control) as the between-subject factor (**Table 2**). A significant two-way interaction would indicate the presence of a training effect that is selective for the training group compared to the control group. The two-way interaction between time and group was significant only for numerosity comparison accuracy [*F*(1,44) = 7.47; *p* < 0.01, *partial* *η*^2^ = 0.15, **Figure 7**, **Table 2**]. (No other interaction effects were significant (*p*s > 0.05; see **Supplementary Table S7** for results of the mixed repeated measures ANOVAs on other measures of basic number processing abilities). Given the significant two-way interaction effect, *post hoc* tests of the simple main effects of group at each time (pre-training, post-training) were conducted for numerosity comparison accuracy. A significant training effect should be manifested as higher post-training (but not pre-training) performance of the training group compared to the control group. There was no difference in numerosity comparison accuracy between the training and control group at pre-training, but the training group had significantly higher numerosity comparison accuracy at post-training [pre-training: *t*(44) = 1.30, *p* = 0.20, post-training: *t*(44) = 4.37, *p* < 0.001, **Figure 7**, **Table 1**]. In other words, training with the “Gathering Ingredients” module improved the training group’s numerosity comparison accuracy.

**Table 2:** The result of mixed 2 × 2 repeated measures ANOVA on numerosity comparison accuracy with group as the between-subject factor and time as the within-subject factor.

Dependent variable		Source	SS	*df*	MS	*F*	*P*	η^2^
Numerosity comparison accuracy	Within subjects	Time	0.02	1	0.02	4.05	0.05	0.08
		Group × time	0.03	1	0.03	7.47	0.01	0.15
		Error	0.16	44	0.01			
	Between subjects	Group	0.10	1	0.10	11.01	0.01	0.20
		Error	0.40	44	0.01			

### Transfer Effects to Math Achievement

Descriptive statistics of performance from pre- and post-training assessments of math achievement are provided in **Table 3**. The results of independent samples *t*-tests at each time (pre-training, post-training) for each assessment score are also shown in **Table 3**. In order to investigate whether the effect of training transfers to improvement on math achievement, a mixed repeated measures ANOVA was conducted on all mathematical achievement scores (KNISE-BAAT and computerized arithmetic) with time (pre-, post-training) as the within-subject factor and group (training, control) as the between-subject factor (**Table 4**). There were no significant interaction effects on either KNISE BAAT or computerized arithmetic scores (*p*s > 0.05, **Table 4**; see **Supplementary Table S7** for results of the mixed repeated measures ANOVA on all other measures of math achievement).

**Table 3:** Pre- and post-training assessment of math achievement (computerized arithmetic, KNISE-BAAT) of the training and control groups.

		Pre-training			Post-training		
		Training	Control	*t*-test	Training	Control	*t*-test
Computerized arithmetic	Accuracy	0.89 (0.13)	0.88 (0.17)	0.322 (*p* = 0.75)	0.92 (0.06)	0.86 (0.15)	1.86 (*p* = 0.07)
	Reaction time	4,505.18 (1,174.94)	6,418.37 (5,249.26)	−1.67 (*p* = 0.10)	4,158.39 (1,673.01)	5,442.65 (3,251.20)	−1.66 (*p* = 0.10)
KNISE-BAAT	Number concept	13.50 (2.39)	12.54 (2.47)	1.34 (*p* = 0.19)	15.32 (2.64)	14.33 (3.07)	1.16 (*p* = 0.25)
	Geometry	13.55 (3.50)	13.88 (3.27)	−0.33 (*p* = 0.74)	14.41 (2.63)	14.96 (2.96)	−0.66 (*p* = 0.51)
	Arithmetic	15.68 (3.03)	14.29 (3.54)	1.42 (*p* = 0.16)	17.23 (2.84)	15.92 (2.62)	1.63 (*p* = 0.11)
	Problem solving	14.68 (4.34)	14.04 (4.60)	0.48 (*p* = 0.63)	17.23 (3.58)	16.79 (3.76)	0.40 (*p* = 0.69)
	Total score	57.41 (9.45)	54.75 (11.31)	0.86 (*p* = 0.39)	64.18 (9.02)	61.92 (10.18)	0.80 (*p* = 0.43)

*Numbers in parentheses denote SDs. The results of between-group t tests at each time (pre-training, post-training) for each assessment score are also shown.*

## Discussion

The present study examined whether or which basic numerical processing ability can be improved with training and whether this training effect can be transferred to improvement in different domains of mathematical achievement. We developed a child-friendly training program called “123 Bakery” which included four training modules (“Gathering Ingredients,” “Guess How Many?,” “Cake Decoration,” and “Selling Cakes”). The dot arrays used as stimuli representing non-symbolic magnitude were controlled so that the influence of non-numerical visual properties was minimized. Exact, symbolic calculation was purposefully excluded from training in order to examine whether training on basic numerical ability improves exact, symbolic calculation while ruling out direct practice or test–retest effects. All participants were assessed on their basic numerical ability twice, 6 weeks apart. The training group participated in 6 weeks of training immediately after the first assessment. The second assessment took place immediately after the training session ended. Compared to the control group, the numerosity comparison accuracy of the training group improved significantly more at post-training assessment. This result is consistent with previous studies reporting improvement of ANS acuity after training (DeWind and Brannon, 2012; Odic et al., 2014; Wang et al., 2016). However, the Training group did not show any greater improvement in math achievement scores compared to the control group. The absence of transfer effect to symbolic math ability after training is consistent with some previous reports (Räsänen et al., 2009; Wilson et al., 2009).

Several aspects of the present study are worth noting. Compared to previous studies, the period of training was much longer and the range of magnitudes used for both training and assessment was much larger. Furthermore, non-numerical visual magnitudes of stimuli were controlled for during both training and assessment. In addition, different visual interfaces of tasks were used between training vs. assessment to prevent direct practice effects. Our training was conducted in the child’s home to improve ecological validity to real-world, educational applications. Based on the analysis of our data, we could not find any evidence in support of training effects that transfer to improvement in any domain of math achievement. The only effect of training observed was improvement in the accuracy of numerosity comparison.

**Table 4:** The result of mixed 2 × 2 repeated measures ANOVA on math achievement scores (computerized arithmetic, KNISE-BAAT) with group as between-subject factor and time as within-subject factor.

Dependent variable		Source	SS	*df*	MS	*F*	*P*	η^2^
Computerized arithmetic (accuracy)	Within subjects	Time	0.01	1	0.01	0.22	0.64	0.01
		Group×time	0.01	1	0.01	1.82	0.19	0.04
		Error	0.33	44	0.01			
	Between subjects	Group	0.04	1	0.04	1.18	0.28	0.03
		Error	1.31	44	0.03			
KNISE-BAAT (total score)	Within subjects	Time	1,115.15	1	1,115.15	44.34	0.01	0.50
		Group×time	0.89	1	0.89	0.04	0.85	0.01
		Error	1,106.60	44	25.15			
	Between subjects	Group	139.16	1	139.16	0.79	0.38	0.01
		Error	7,804.32	44	177.37			

*The total score of KNISE-BAAT represents the mean of all subtests.*

### Comparison With Other Training Studies

The results of the present study are in contrast with those reported by some training studies conducted with young children (Wilson et al., 2009; Obersteiner et al., 2013; Hyde et al., 2014; Khanum et al., 2016; Maertens et al., 2016; Park et al., 2016; Sella et al., 2016; Wang et al., 2016). In our study, only non-symbolic numerosity comparison performance (but not PAE from symbolic numberline estimation) improved after training without any transfer effects to math achievement. In contrast, Maertens et al. (2016) reported that only the PAE of numberline estimation (but not numerosity comparison) improved significantly more in the training compared to the control group, but both training effects transferred to improvement on pictorially presented (but not symbolic) arithmetic problems in preschoolers. In Hyde et al. (2014), single session practice on both approximate addition and numerosity comparison (but not line length addition or brightness comparison) led to gains in exact, symbolic addition (but not sentence comparison) (Hyde et al., 2014). In Wang et al. (2016), 5-year-old children who were briefly trained to improve their precision of numerosity discrimination showed higher performance on symbolic math (but not vocabulary) compared to the control group. In this study, improvement in children’s ANS acuity was brought about by presenting trials in “easy to hard” order, to induce the experience of a sequence of confident problem solving (“confidence hysteresis”) which the authors believe leads to enhancement of ability (Odic et al., 2014). In the control groups, trials were presented in the opposite or random orders. However, there were no pre-training assessment of ANS acuity or math ability in Wang et al., (2016), thus it is difficult to rule out pre-training differences in ANS acuity or math ability between groups. Furthermore, some researchers question whether the transfer effects observed in Hyde et al. (2014) or Wang et al. (2016) reflect attentional priming to numerical representations rather than true transfer effects to math improvement, given the brevity of the practices in these two studies (Szűcs and Myers, 2017). Taken together, although it is not possible to definitively state the cause of these discrepancies, possible sources may include differences in how pre- and post-training assessments were made and the duration of training. The present study conducted pre- and post-training assessment of all abilities included in the training program using a separate task designed with a different visual interface. Thus, in the present study, mere practice (or test–retest) effects were minimized in the post-training assessment, making it less likely to see improvement on the outcome ability. It is also possible that in young children, ANS performance is facilitated by the presence of non-numerical visual magnitudes that are correlated with numerosity (Defever et al., 2013; Szucs et al., 2013). The present study controlled for the influence of non-numerical visual properties of the stimuli during both training and assessment. Differences in the method by which non-numerical visual magnitudes were controlled across studies may have influenced the discrepancy in the type of cognitive process trained and the resulting assessment of outcome ability. Considering the observation of Szkudlarek and Brannon (2018), it should also be emphasized that individuals (especially young children) with low ability may benefit more from approximate arithmetic training and that transfer effects of training may be specific to certain domains or components of math ability (e.g., informal math skills as opposed to formal math skills).

### Factors that May Influence Transfer Effects

Inconsistencies across studies may also be due to differences in the contents of the training across studies. First of all, transfer effects to symbolic math reported from training studies which included symbolic arithmetic practice (e.g., Number Race or Rescue Calcularis) may reflect direct practice effects because the training program itself included symbolic arithmetic practice (Wilson et al., 2006, 2009; Vilette et al., 2010; Kucian et al., 2011; Obersteiner et al., 2013; Sella et al., 2016). Second, training may be less effective when multiple modules are included within a single session. Several training studies which involved practicing a single type of process (e.g., approximate arithmetic, numerosity comparison, or number line estimation) observed significant transfer effects (Park and Brannon, 2013; Hyde et al., 2014; Park and Brannon, 2014; Khanum et al., 2016; Maertens et al., 2016; Park et al., 2016; Au et al., 2018). In contrast, when training involves multiple kinds of training modules (as in our study), transfer effects may be less easily observed, due to increased variability in the effectiveness of each training module across participants. For example, some participants may be relatively more engaged and motivated by module A, while others by module B, and so forth. In such cases, the group average of the training effect of each module may be reduced by increased individual variability and (by the same reason) the resulting transfer effect may also be washed out, especially if each module is more or less associated with partially different components of the outcome ability. Thus, increased variability in training effects of each module across individuals may have caused the absence of direct improvement on some of the trained tasks (numberline estimation or approximate arithmetic ability, etc.) in the present study, especially given the small sample size.

Based on the observation that training on approximate arithmetic but not numerosity comparison transfers to improvement on symbolic arithmetic in adults, Park and Brannon (2014) hypothesized that cognitive training may have positive transfer effects if training and the outcome ability share *common mental operations* (Park and Brannon, 2014). Alternatively, Hyde et al. (2016) hypothesized that transfer effects may be determined by the *overlap of mental representations* between training and the outcome ability (at least in children). This hypothesis was based on the observation that training effects from both numerosity comparison and approximate addition transferred to improvement in symbolic addition in children (Hyde et al., 2016). The absence of transfer effect to symbolic math ability despite improved ANS acuity in the present study seems to support the idea that transfer effects of training require substantial overlap of mental operations between the trained process and the outcome ability, consistent with Park & Brannon’s “Operational Overlap” hypothesis. Taken together, as Hyde et al. (2016) had mentioned as well, we emphasize that finding the answer to the question of which type of basic mathematical training can enhance mathematical cognition requires continued efforts, taking into consideration that factors such as developmental changes and subtle differences in research methodology can critically influence this relationship.

### Limitations and Directions for the Future

We acknowledge that it would been better to include another kind of active training (unrelated to basic numerical processing) for the control group. We acknowledge this as a limitation of the present study. If there had been a transfer effect of training on math achievement selectively for the training group, it would have been hard to eliminate the possibility of a placebo (or Hawthorne) effect. However, given the absence of a transfer effect, the lack of a control training program can be thought to be less of a problem in the case of the present study. Furthermore, Szűcs and Myers (2017) emphasizes that it is not meaningful to contrast target-related interventions with target-irrelevant ones (e.g., contrasting math training vs. reading or drawing interventions) or to contrast between two interventions which are not equally engaging, motivating, and intellectually stimulating. Although the present study lacks an active control group, we can at least contrast the efficacy of different types of basic math training based on a within-subjects design, while all training can be considered to be equally engaging and motivating. [All submodules were based on a coherent theme (i.e., animals baking cake, animals selling cake, etc.), user interface (presentation of colorful cartoons with music), and method of feedback.] Regardless, in future studies, it would be ideal to contrast the effect of cognitive training in the experimental group against a well-matched, alternative form of training for the control group.

The absence of transfer effect in our home-based training study raises the question of whether transfer effects observed within a lab-based setting will generalize to real-world or actual educational applications. Taken together, the results of the present study reveal that (1) only certain kinds of basic numerical ability (in the present case, only ANS acuity) of young children can be improved with training and (2) improvement on ANS acuity does not seem transfer to improvement in math achievement, despite extensive training for 6 weeks, across large ranges of magnitudes.

## Data Availability Statement

The raw data supporting the conclusions of this manuscript will be made available by the authors, without undue reservation, to any qualified researcher.

## Author Contributions

NK contributed to designing the pre- and post-training assessments, improving the design of the training program, data collection, and analysis. SJ contributed to designing the training paradigm, data collection, and analysis. SC is the principle investigator who contributed to all aspects of the study from acquiring funding, designing the study, and monitoring all aspects of data collection and analysis. All authors contributed to writing the manuscript.

## Conflict of Interest Statement

The authors declare that the research was conducted in the absence of any commercial or financial relationships that could be construed as a potential conflict of interest.

## Footnotes

1 The Number Race program includes numerosity comparison, mapping between symbolic and non-symbolic number, and symbolic addition/subtraction.
