# Supplementary material for: Testing the Efficacy of Training Basic Numerical Cognition and Transfer Effects to Improvement in Children’s Math Ability
Source: Front Psychol. 2018 Oct 2;9:1775. doi: 10.3389/fpsyg.2018.01775 (PMC6175973; doi:10.3389/fpsyg.2018.01775)
Supplement: Supplementary file 1 [file Data_Sheet_1.pdf]

Supplementary Materials

Testing the Efficacy of Training Basic Numerical Cognition and Transfer Effects to Improvement in Children's Math Ability

Narae Kim, Selim Jang and Soohyun Cho*

***Corresponding Author: soohyun@cau.ac.kr

1 Supplementary Methods

1.1. Data Quality Control

Data from 5 children who did not complete the experiment and 5 children whose performance was lower than 2SDs below the mean were excluded. Among the 5 children, 2 were excluded for poor performance in terms of the final level reached from the training modules, and 3 were excluded due to poor performance in terms of the pre-training numerosity comparison performance.

Because of the large number of assessments involved, we used the following three scores for quality control; 1) the final level reached for Gathering Ingredients, 2) the final level reached for Guess How Many, 3) pre-training numerosity comparison accuracy.

The cut-off thresholds for the final level reached for Gathering Ingredients was 42.29, for Guess How Many 13.49 and for pre-training numerosity comparison accuracy 0.55. One participant's final level reached for Gathering Ingredients was 38. Another participant's final level reached for Guess How Many was 6. These two participants were excluded because their performance was less than the thresholds. Also, 3 participants' pre-training numerosity comparison accuracy were .45, .53, .3, respectively, which were below the cut-off threshold.

1.2 Specification of stimuli for each Level of '123 Bakery' and required performance for Level upgrades

In all modules of '123 Bakery', the magnitudes (i.e., set size and number size) increased and the ratio of magnitudes approached 1, as the Level increased. The required level of performance to upgrade to the next Level increased as well, as the Level increased (i.e., it became more and more difficult to upgrade to the next Level). In order to upgrade to the next Level, participants had to achieve a certain degree of accuracy (hereafter, reference accuracy) within a certain number of recent trials (hereafter, reference trials). The number of reference trials also increased as the Level increased. The first Level of each module was for practice.

Table S1. Training Module 1: Numerosity Comparison ('Gathering Ingredients')

Level	Numerosity Range	Numerosity Ratio	Reference Accuracy (proportion correct)	Number of reference trials
1	6~15	2:3	100%	3
2	6~15		90%	10
3	10~30			
4	30~50			
5	50~100			
6	100~200			
8	6~15	3:4		
9	10~30			
10	30~50			
11	50~100			
12	100~200			
14	6~15	4:5		15
15	15~30			
16	30~50			
17	50~100			
18	100~200			
20	6~15	5:6		
21	10~30			
22	30~50			
23	50~100			
24	100~200			
26	6~15	6:7	85%	20
27	10~30			
28	30~50			
29	50~100			
30	100~200			
32	6~15	7:8		
33	10~30			
34	30~50			
35	50~100			
36	100~200			
38	6~15	8:9		

39	10~30			
40	30~50			
41	50~100			
42	100~200			
44	10~30	9:10		
45	30~50			
46	50~100			
47	100~200			
48	200~300			
49	10~30	10:11		
50	30~50			
51	50~100			
52	100~200			
53	0~100	8:9		
54	50~200	8:9		
55	0~100	3:4~8:9	90%	
56	50~200	3:4~8:9		
57	0~100	6:7~8:9		
58	50~200	7:6~9:8		
59	6~20	9:10	85%	
60	20~40			
61	40~60			
62	60~100			
63	100~200			
64	0~100			
65	50~200			
66	0~25	10:11		
67	20~45			
68	40~70			
69	70~100			
70	100~200			
71	0~100			
72	70~200			
73	0~100	4:5~10:11	90%	
74	50~200	3:4~10:11		
75	0~100	6:7~10:11		
76	50~200	6:7~10:11		
77	0~100	8:9~10:11		

78	50~200	8:9~10:11		
79	0~200	3:4~10:11		
80	0~200	6:7~10:11		
81	0~200	8:9~10:11		

Table S2. Training Module 2: Non-symbolic numberline estimation ('Guess How Many?')

Trials in which the participant localized the estimate within the 'accurate zone' were counted as accurate trials. The relative width of the accurate zone (calculated as a % relative to the width of the entire numberline) decreased as the Level increased.

Level	Numerosity Range	The relative width of the accurate zone (%)	Reference Accuracy	Number of Reference trials
1	5~10	30%	60%	3
2	5~10			5
3	5~10			10
4	0~10		75%	12
5	10~30			
6	5~30			
7	0~30			
8	30~50			
9	0~50			
10	50~80			
11	0~80			
12	0~80			
13	0~80	25%		
14	75~100	30%		
15	30~100			
16	0~100			
17	0~100			
18	0~100	25%		
19	100~150	30%		
20	50~150			
21	0~150			
22	0~150			
23	0~150	25%		

24	150~200	30%		
25	75~200			
26	0~200			
27	0~200			
28	0~200	25%		
29	0~80	20%		
30	0~80			
31	0~100			
32	0~100			
33	0~150			
34	0~150			
35	0~200			
36	0~200			

Table S3. Training Module 3: Non-symbolic addition/subtraction ('Cake Decoration')

Level	Numerosity Range	Numerosity Ratio	Reference Accuracy	Number of Reference trials
1	0~50	1:3	60%	3
2	0~50			5
3	0~50			10
4	0~50		75%	12
5	0~60			
6	0~80			
7	0~100			
8	0~30	1:2		
9	0~40			
10	0~60			
11	0~110			
12	0~30	2:3		
13	0~40			
14	0~60			
15	0~110			
16	0~30	3:4		
17	0~40			
18	0~50			
19	0~110			

20	0~30	4:5		
21	0~40			
22	0~60			
23	0~110			
24	0~30	5:6		
25	0~50			
26	0~60			
27	0~110			
28	0~30	6:7		
29	0~40			
30	0~60			
31	0~110			
32	0~30	7:8		
33	0~40			
34	0~60			
35	0~110			
36	0~30	8:9		
37	0~40			
38	0~60			
39	0~110			

Table S4. Training Module 4: Symbol-to-Numerosity Mapping (‘Selling Cakes’)

Level	Numerosity Range	Numerosity Ratio	Reference Accuracy	Number of Reference trials
1	6~15	2:3	60%	3
2	6~15			5
3	6~15			10
4	6~15		75%	12
5	10~30			
6	30~50			
7	50~100			
8	100~200			
9	0~100			
10	50~200			
11	6~15	3:4		

12	10~30			
13	30~50			
14	50~100			
15	100~200			
16	0~100			
17	50~200			
18	6~15			
19	10~30			
20	30~50			
21	50~100	4:5		
22	100~200			
23	0~100			
24	50~200			
25	6~15			
26	10~30			
27	30~50			
28	50~100	5:6		
29	100~200			
30	0~100			
31	50~200			
32	0~100	3:4~5:6	85%	
33	50~200			
34	6~15			
35	10~30			
36	30~50			
37	50~100	6:7		
38	100~200			
39	0~100			
40	50~200			
41	6~20		75%	
42	20~40			
43	30~50			
44	50~100	7:8		
45	100~200			
46	0~100			
47	60~200			
48	6~20	8:9		

<table>
<tr><td>49</td><td>20~40</td><td rowspan="6"></td><td rowspan="6"></td><td rowspan="10"></td></tr>
<tr><td>50</td><td>40~60</td></tr>
<tr><td>51</td><td>60~100</td></tr>
<tr><td>52</td><td>100~200</td></tr>
<tr><td>53</td><td>0~100</td></tr>
<tr><td>54</td><td>60~200</td></tr>
<tr><td>55</td><td>0~100</td><td rowspan="3">6:7~8:9</td><td rowspan="4">90%</td></tr>
<tr><td>56</td><td>50~200</td></tr>
<tr><td>57</td><td>0~100</td></tr>
<tr><td>58</td><td>0~200</td><td>3:4~8:9</td></tr>
</table>

1.3 Specification of stimuli for the numerosity comparison task (used for pre- and post-training assessment).

Table S5. Specification of stimuli for the numerosity comparison task. Trials were evenly divided into Area-controlled and Size-controlled conditions.

**Magnitude ranges**	**Ratio of magnitudes**					
	**1:2**	**3:4**	**5:6**	**6:7**	**7:8**	**8:9**
**0-20**	6:12	9:12	12:10	7:6	8:7	9:8
**20-30**	15:30	21:28	30:25	28:24	24:21	27:24
**30-50**	25:50	26:48	48:40	49:42	48:42	45:40
**50-100**	50:100	75:100	96:80	98:84	96:84	99:88
**100-200**	100:200	150:200	198:165	196:168	200:175	198:176
**Size-controlled condition**	10	10	10	10	10	10
**Area-controlled condition**	10	10	10	10	10	10
**Total number of trials**	20	20	20	20	20	20

2 Supplementary Results

Table S6. Descriptive statistics of performance on each module of '123 Bakery'.

The final level refers to the final level reached at the end of training (at the end of the 30th session). Mean accuracy and reaction time refers to the overall average of the 30 mean performance scores (accuracy and RT) from each session.

	Final Level	Mean Accuracy	Mean Reaction time
**Gathering Ingredients**	57.73(7.72)	77.34(5.74)	1293.92(170.99)
**Guess How many?**	25.73(6.11)	0.14(0.02)#	1463.85(272.44)
**Cake Decoration (Addition)**	42.18(2.46)	63.07(11.38)	1820.66(462.72)
**Cake Decoration(Subtraction)**	40.55(2.89)	51.63(3.12)	1900.81(483.477)
**Selling Cakes**	41.63(8.66)	55.61(3.34)	1796.28(239.27)

PAE (Percentage Absolute Error)

Table S7. The result of mixed repeated measures ANOVA with Group as the between-subject factor and Time as the within-subject factor on all measures of basic numerical processing abilities and math achievement (except for those included in Tables 2 and 4).

DependentVariable		Source	SS	df	MS	F	P	η^2
**Computerized Arithmetic RT**	**Within Subjects**	**Time**	1.004e+7	1	1.004e+7	1.05	.31	.02
		**Group × Time**	2.27e+6	1	2.27e+6	.24	.63	.01
		**Error**	4.21e+8	44	9.58e+6			
	**Between subjects**	**Group**	5.87e+7	1	5.87e+7	4.75	.04	.10
		**Error**	5.43e+8	44	1.24e+7			
**KNISE-BAAT Number Concept**	**Within Subjects**	**Time**	74.79	1	74.79	32.38	<.001	.42
		**Group × Time**	.004	1	.004	.002	.97	<.001
		**Error**	101.62	44				
	**Between subjects**	**Group**	21.67	1	21.67	1.83	.18	.04
		**Error**	521.95	44	11.86			

**KNISE-BAAT Geometry**	**Within Subjects**	**Time**	21.76	1	21.76	5.69	.02	.11
		**Group × Time**	.28	1	.28	.07	.79	.001
		**Error**	168.21	44	3.82			
	**Between subjects**	**Group**	4.43	1	4.432	.29	.60	.006
		**Error**	682.14	44	15.503			
**KNISE-BAAT Arithmetic**	**Within Subjects**	**Time**	57.69	1	57.69	20.71	<.001	.32
		**Group × Time**	.04	1	.04	.01	.91	<.001
		**Error**	122.54	44	2.79			
	**Between subjects**	**Group**	41.86	1	41.86	2.68	.11	.06
		**Error**	686.89	44	15.61			
**KNISE-BAAT Problem Solving**	**Within Subjects**	**Time**	160.94	1	160.94	18.16	<.001	.29
		**Group × Time**	.24	1	.24	.027	.87	<.001
		**Error**	389.98	44	8.86			
	**Between subjects**	**Group**	6.64	1	6.64	.27	.61	.006
		**Error**	1087.58	44	24.72			
**Numerosity Comparison RT**	**Within Subjects**	**Time**	258958	1	258958	4.48	.04	.09
		**Group× Time**	28192	1	28192	.49	.49	.01
		**Error**	2.55e+6	44	57870			
	**Between subjects**	**Group**	126.20	1	126.20	9.16e-4	.98	<.001
		**Error**	6.10e+6	44	137787			
**Symbolic Numberline Estimation PAE**	**Within Subjects**	**Time**	1.42e-4	1	1.42e-4	.74	.39	.02
		**Group × Time**	1.76e-4	1	1.76e-4	.93	.34	.02
		**Error**	.008	44	1.91e-4			
	**Between subjects**	**Group**	3.82e-4	1	3.82e-4	.84	.36	.02
		**Error**	.02	44	4.39e-4			

**Symbolic Numberline Estimation RT**	**Within Subjects**	**Time**	8.79	1	8.79	10.17	.003	.19
		**Group × Time**	3.27	1	3.27	3.78	.06	.08
		**Error**	38.03	44	.86			
	**Between subjects**	**Group**	5.48	1	5.48	3.80	.06	.08
		**Error**	63.54	44	1.44			
**Non-symbolic Numberline Estimation PAE**	**Within Subjects**	**Time**	.001	1	.001	1.16	.29	.03
		**Group × Time**	.003	1	.003	2.49	.12	.06
		**Error**	.055	44	.001			
	**Between subjects**	**Group**	.002	1	.002	1.29	.26	.03
		**Error**	.06	44	.001			
**Non-symbolic Numberline Estimation RT**	**Within Subjects**	**Time**	.24	1	.24	.41	.52	.01
		**Group × Time**	.004	1	.004	.007	.93	1.55e-4
		**Error**	24.47	44	.57			
	**Between subjects**	**Group**	.33	1	.33	.25	.62	.006
		**Error**	55.31	44	1.29			
**Non-symbolic Addition ACC**	**Within Subjects**	**Time**	.003	1	.003	.39	.53	.01
		**Group × Time**	.01	1	.01	1.35	.25	.03
		**Error**	.29	44	.01			
	**Between subjects**	**Group**	.01	1	.07	.85	.36	.02
		**Error**	.36	44	.01			
**Non-symbolic Addition RT**	**Within Subjects**	**Time**	4409	1	4409	.01	.93	<.001
		**Group × Time**	270241	1	270241	.47	.50	.01
		**Error**	2.53e+7	44	575099			
	**Between subjects**	**Group**	156455	1	156455	.21	.65	.01
		**Error**	3.36e+7	44	763426			

**Non-symbolic Subtraction ACC**	**Within Subjects**	**Time**	.02	1	.02	3.03	.09	.06
		**Group × Time**	.001	1	.001	.20	.66	.004
		**Error**	.24	44	.01			
	**Between subjects**	**Group**	.01	1	.01	1.10	.30	.02
		**Error**	.29	44	.01			
**Non-symbolic Subtraction RT**	**Within Subjects**	**Time**	2.26e+6	1	2.26e+6	1.39	.24	.03
		**Group × Time**	79864	1	79864	.05	.83	.001
		**Error**	7.13e+7	44	1.62e+6			
	**Between subjects**	**Group**	1.21e+6	1	1.21e+6	.63	.43	.01
		**Error**	8.52e+7	44	1.94e+6			
**Symbol-to-Numerosity Mapping ACC**	**Within Subjects**	**Time**	3.85e-4	1	3.85e-4	.13	.72	.003
		**Group × Time**	.003	1	.003	.89	.35	.02
		**Error**	.13	44	.003			
	**Between subjects**	**Group**	.01	1	.01	1.01	.32	.02
		**Error**	.22	44	.01			
**Symbol-to-Numerosity Mapping RT**	**Within Subjects**	**Time**	50208	1	50208	.28	.60	.01
		**Group × Time**	189644	1	189644	1.08	.31	.02
		**Error**	7.78e+6	44	176696			
	**Between subjects**	**Group**	1.62e+6	1	1.62e+6	3.92	.05	.08
		**Error**	1.81e+7	44	411762			
